# Incidence of Gastric Adenocarcinoma in Those With Gastric Atrophy: A Systematic Review

**DOI:** 10.7759/cureus.71768

**Published:** 2024-10-18

**Authors:** Eoghan Burke, Patricia Harkins, Mayilone Arumugasamy

**Affiliations:** 1 Surgery, Royal College of Surgeons in Ireland (RCSI), Dublin, IRL; 2 Medicine, Royal College of Physicians of Ireland (RCPI), Dublin, IRL

**Keywords:** atrophic gastritis, gastric atrophy, gastric cancer, pre-neoplastic, standardised incidence ratios

## Abstract

Gastric atrophy (GA), or atrophic gastritis, is a pre-neoplastic lesion of gastric cancer (GC). It is part of the Correa cascade, which culminates in intestinal-type gastric adenocarcinoma. The cascade posits that intestinal-type gastric adenocarcinoma develops along a defined pathway of pre-neoplastic stages. The cascade begins with chronic gastritis, most commonly caused by Helicobacter pylori (H. pylori) infection, and proceeds through GA, gastric intestinal metaplasia (GIM), both complete and incomplete, dysplasia, both low and high-grade, and culminating in intestinal-type gastric adenocarcinoma. Attempts in Europe have been made to identify patients at risk of developing GC and target them with surveillance oesophagogastroduodenoscopy (OGD). However, there remains uncertainty about GA's risk of developing into GC. This poses issues in terms of guiding the need for and determining intervals for surveillance OGDs, which are a costly form of surveillance. As such, we attempted to gather all available studies assessing the risk of GC developing from GA, which is the first step in the Correa cascade. This study was a comprehensive systematic review of published papers, reported per the Preferred Reporting Items for Systematic Reviews and Meta-Analyses (PRISMA) statement.

This systematic review, which included a substantial 25,455 patients across 18 studies, found that the relative risk (RR) of GC in those with GA, using standardised incidence ratios as a measure of RR, was 15.1, with a 95% confidence interval ranging from 13.5 to 16.9. We conclude that GA does increase the risk of developing GC, and this risk may be higher than previously appreciated. Further large-scale studies are needed in Western cohorts of patients to precisely define this risk and guide the need for surveillance programs. These future studies must be standardised to account for H. pylori status, the topographical distribution of the GA, and the methods for assessing the degree of GA.

## Introduction and background

Gastric atrophy (GA), or atrophic gastritis, is a pre-neoplastic lesion of gastric cancer (GC). GA is the loss of the normal glandular architecture of the stomach in the setting of chronic gastritis [1]. It is part of the Correa cascade, which culminates in intestinal-type gastric adenocarcinoma. The cascade posits that intestinal-type gastric adenocarcinoma develops along a defined pathway of pre-neoplastic stages. The cascade begins with chronic gastritis, most commonly caused by Helicobacter pylori (H. pylori) infection or autoimmune gastritis, and proceeds through GA, gastric intestinal metaplasia (GIM), both complete and incomplete, dysplasia, both low and high grade, and culminating in intestinal-type gastric adenocarcinoma [2]. This finding has led to attempts to identify patients at early stages in this cascade to target them with surveillance programs to identify GC at earlier stages.

GC is one of the most common cancers in both sexes and counts as the third leading cause of cancer mortality worldwide [3]. There is significant geographical variation in the prevalence and incidence of GC, with the highest incidence rates reported in eastern countries, including 39.6/100,000 in South Korea [4]. This contrasts with rates of 7.7/100,000 in Sweden [5], which would be representative of the incidence in low-risk western populations. Given the high incidence rates in eastern countries, there have long been surveillance programs in place in these countries in an attempt to identify patients at risk of developing, or those with early, GC.

In Europe, attempts have also been made to identify patients at risk of developing GC and target them with surveillance oesophagogastroduodenoscopy (OGD). The British Society of Gastroenterology and the European Society of Gastroenterology have released guidelines outlining their surveillance programs.

However, there remains uncertainty about GA's risk of developing into GC. This poses issues in guiding the need for and determining intervals for surveillance OGDs, a costly form of surveillance [6].

As such, we attempted to gather all available studies assessing the risk of GC developing from GA, which is the first step in the Correa cascade.

## Review

Materials and methods

Study Aims and Objectives

Our study aimed to synthesise the available evidence on the incidence of GC in those with GA.

Study Design

This study was a systematic review of published papers, which is reported per the Preferred Reporting Items for Systematic Reviews and Meta-Analyses (PRISMA) statement [7].

Eligibility Criteria

The inclusion criteria for our study comprised all observational studies which assessed the risk of GC in those with GA. We included both retrospective and prospective studies. All studies included were in the English language and involved adult patients. Studies not meeting these criteria were excluded.

Search Strategy

A detailed search strategy was developed following literature review. Keywords and MeSH terms relating to GC and GA were used to create the search string: ((gastric cancer) OR (gastric adenocarcinoma) OR (Gastric carcinoma)) AND ((gastric atrophy) OR (Atrophic gastritis)). This search string was then applied to the following bibliographic databases: PubMed, EMBASE and the Cochrane Central Register of Controlled Trials (CENTRAL). We used Google Scholar to conduct a citation search during the study selection process, as described below. This ensured that we were unlikely to miss relevant studies while limiting the number of initial results screened. All databases were searched from 2010 to the present day to identify the most recent studies in this area, acknowledging changing trends in risk factors over time [8].

A systematic review manager was used for this study [9].

Study Selection

After removing duplicates, two authors independently screened all identified studies’ titles and abstracts. Abstracts meeting the previously described inclusion criteria were selected. If there was any conflict about a study’s inclusion, this was resolved by a third author. The resulting studies were reviewed in full, and eligibility for inclusion in qualitative analysis was determined. Any conflict about a study’s eligibility was resolved with consensus. During the article review, a hand search of references was conducted to identify any studies not identified in the original search. Similarly, a citation search using Google Scholar on all eligible articles was completed again to ensure no further studies were omitted.

Data Extraction

Two authors independently extracted data from the selected studies using a predetermined data extraction form. The data extracted included the study's title, authors, year of publication, country of origin, study design, patient demographics, number of patients with GA, number of patients with GC, follow-up period, H. pylori status, and assessment method of GA. Further details about a study's description of the topographical distribution of the GA were also extracted.

Statistical Analysis

Incidence rates were calculated per 1000 person-years. Standardised incidence ratios (SIRs) and 95% confidence intervals were calculated as measures of relative risk. They were calculated by comparing the rate of observed cases of GC in the GA cohorts versus the expected rate of GC in the general population for the country of origin of the study. The expected rates were obtained from the WHO's Global Cancer Observatory database [10].

Results

Study Selection

**Figure 1:**
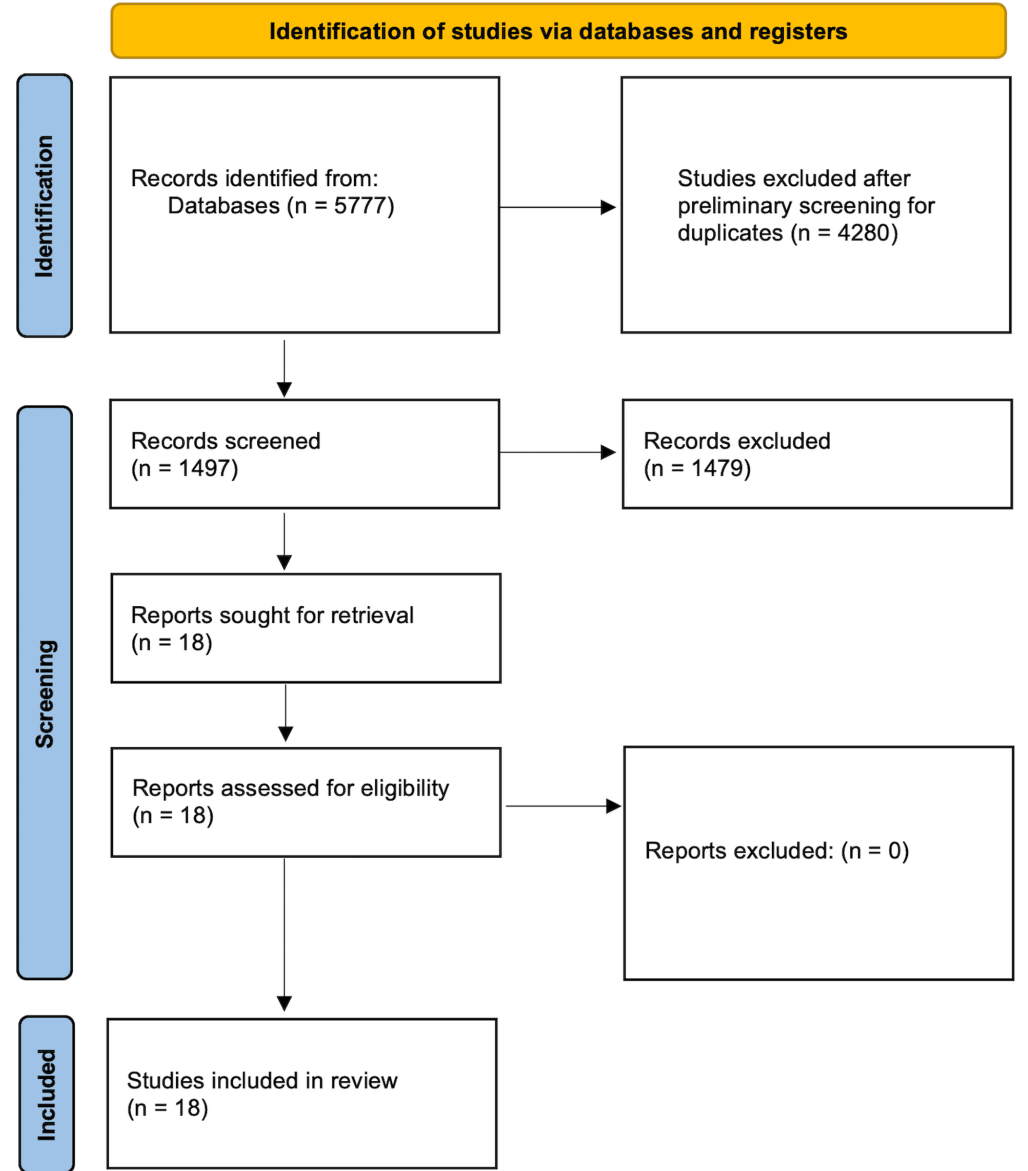
Preferred Reporting Items for Systematic Reviews and Meta-Analyses (PRISMA) flowchart. This depicts the selection of studies for systematic review.

The number of articles found via searching the bibliographic databases PubMed, EMBASE, and The Cochrane Central Register of Controlled Trials was 5777. After removing duplicates, the number of original articles to screen was 1497. These articles' titles and abstracts were screened independently by two authors as described. Articles meeting criteria for further evaluation of full text numbered 18. These studies were included in the final review for narrative synthesis (Figure 1).

Study Characteristics

Table 1 details the characteristics of the included studies. This systematic review included 18 studies comprising 25,455 patients. The included studies comprised 10 retrospective cohort studies and eight prospective studies. Seven studies were conducted in Eastern countries, while 11 were conducted in Western countries. The patient numbers enrolled in the studies varied from a low of five to a high of 14285.

**Table 1:** Summary of study characteristics.

Study ID	Study Design	Year of Publication	Country	Number of patients with Gastric Atrophy	Mean Age
Take et al [11]	Retrospective cohort study	2020	Japan	2737	53.8
Zhang et al [12]	Retrospective cohort study	2018	China	332	63
Song et al [13]	Retrospective cohort study	2017	South Korea	2144	47.8
Toyoshima et al [14]	Retrospective cohort study	2017	Japan	1194	54.1
Sekikawa et al [15]	Retrospective cohort study	2016	Japan	847	Not reported
Boreiri et al [16]	Prospective cohort study	2013	Iran	333	52.7
Chen et al [17]	Retrospective cohort study	2012	China	1592	Not reported
Miceli et al [18]	Prospective cohort study	2019	Italy	282	60.3
Chapelle et al [19]	Retrospective cohort study	2020	France	5	61
Rugge et al [20]	Prospective cohort study	2010	Italy	35	55
Esposito et al [21]	Retrospective cohort study	2021	Italy	160	65.7
Hollander et al [22]	Prospective cohort study	2018	Netherlands	11	57.9
Nieminen et al [23]	Prospective cohort study	2019	Finland	1137	78
Dilaghi et al [24]	Prospective cohort study	2018	Italy	80	64.5
Lahner et al [25]	Prospective cohort study	2017	Italy	29	66
Ekheden et al [26]	Retrospective cohort study	2015	Sweden	14285	60.3
Pilozzi et al [27]	Prospective cohort study	2015	Italy	200	55
Segato et al [28]	Retrospective cohort study	2013	Italy	52	56.5

Incidence Rate of GC Cases per 1000 Person-Years in Those With GA

Within the 18 studies, 25,455 patients with GA were followed for 220,014.04 person-years. During this time, 372 GC cases were detected. This results in a pooled incidence rate of 1.7 cases per 1000 person-years with a 95% confidence interval of 1.5 to 1.9 (Table 2).

**Table 2:** The pooled incidence rate of Gastric Cancer cases per 1000 person-years in those with Gastric Atrophy.

Study ID	Number of patients with Gastric Atrophy	Number of Cases of Gastric Cancer	Average Follow-up period in years	Person years	Incidence rate per 1000 person-years	95% CI lower	95% CI upper
Take et al [11]	2737	68	7.1	19,432.70	3.5	2.7	4.4
Zhang et al [12]	332	16	9.2	3054.4	5.2	2.9	8.5
Song et al [13]	2,144	34	6.9	14793.6	2.3	1.6	3.2
Toyoshima et al [14]	1194	15	2.46	2937.24	5.1	2.9	8.4
Sekikawa et al [15]	847	29	5.25	4446.75	6.5	4.4	9.4
Boreiri et al [16]	333	24	9.9	3296.7	7.3	4.7	10.8
Chen et al [17]	1592	23	6	9552	2.4	1.5	3.6
Miceli et al [18]	282	0	3	846	0	0	0
Chapelle et al [19]	5	0	5.5	27.5	0	0	0
Rugge et al [20]	35	2	12.42	434.7	4.6	0.6	16.6
Esposito et al [21]	160	3	3	480	6.3	1.3	18.3
Hollander et al [22]	11	0	4.75	52.25	0	0	0
Nieminen et al [23]	1137	35	13.6	15463.2	2.3	1.6	3.1
Dilaghi et al [24]	80	0	3	240	0	0	0
Lahner et al [25]	29	0	3	87	0	0	0
Ekheden et al [26]	14285	116	10	142850	0.8	0.7	1
Pilozzi et al [27]	200	4	7.5	1500	2.7	0.7	6.8
Segato et al [28]	52	3	10	520	5.8	1.2	16.9
Pooled	25455	372	6.8	220,014.04	1.7	1.5	1.9

Subgroup Analysis of Incidence Rate Grouped by Country of Origin: Eastern Versus Western Countries

Seven studies originated in Eastern countries [11-17]. These seven studies comprised 9179 patients followed for a total of 57,513.39 person-years. Within this cohort, 209 cases of GC were recorded. This yields an incidence rate of 3.6 cases of GC per 1000 person-years with a 95% confidence interval of 3.2 to 4.2 (Table 3).

**Table 3:** Pooled incidence rates of Gastric Cancer in those with Gastric Atrophy per 1000 person-years pooled by region based on country of origin of study.

Region	Number of patients with Gastric Atrophy	Number of Cases of Gastric Cancer	Person years of follow-up	Incidence rate per 1000 person-years	95% CI lower	95% CI upper
Eastern	9179	209	57,513.39	3.6	3.2	4.2
Western	16276	163	162,500.65	1	0.9	1.2

Eleven of the included studies originated in Western countries [18-28]. These 11 studies comprised 16276 patients, and they were followed for a total of 162,500.65 years. During this period, 163 cases of GC were recorded. This yields an incidence rate of one case per 1000 person-years with a 95% confidence interval of 0.9 to 1.2 (Table 3).

Standardised Incidence Ratio (SIR) of GC Cases in GA Cohorts Versus Expected Rates in the General Population

Standardised incidence ratios (SIRs) were used to measure relative risk. They were calculated by comparing the rate of observed cases of GC in the GA cohorts versus the expected rate of GC in the general population for the country of origin of the study. The expected rates were obtained from the WHO's Global Cancer Observatory database [10].

The incidence rates of GC per 1000 person-years for each study were extrapolated to 100,000 person-years to facilitate comparison with expected rates in 100,000 person-years (Table 4).

**Table 4:** Standardised Incidence ratio (SIR) of Gastric Cancer cases in Gastric Atrophy cohorts versus expected rates in the general population.

Study ID	Observed Gastric Cancer incidence rate per 100,000 person-years	Expected incidence rate of Gastric Cancer per 100,000 person-years for country of origin of study	Standardised Incidence Ratio	95% CI Lower	95% CI Upper
Take et al [11]	350	27.5	12.7	11.43	14.13
Zhang et al [12]	520	20.7	25.1	23	27.4
Song et al [13]	230	39.6	5.8	5	6.6
Toyoshima et al [14]	510	27.5	18.6	17	20.2
Sekikawa et al [15]	650	27.5	23.6	21.9	25.5
Boreiri et al [16]	730	21.6	33.8	31.4	36.3
Chen et al [17]	240	20.7	11.6	10.17	13.16
Miceli et al [18]	0	16	0	0	0
Chapelle et al [19]	0	11.5	0	0	0
Rugge et al [20]	460	16	28.7	26.2	31.5
Esposito et al [21]	630	16	39.4	36.4	42.6
Hollander et al [22]	0	10.9	0	0	0
Nieminen et al [23]	230	10	23	20.1	26.2
Dilaghi et al [24]	0	16	0	0	0
Lahner et al [25]	0	16	0	0	0
Ekheden et al [26]	80	7.7	10.4	8.2	12.9
Pilozzi et al [27]	270	16	16.9	14.9	19
Segato et al [28]	580	16	36.25	33.4	39.3

Pooled Global and Regional SIR for GC in Those With GA

The average expected rate of GC for the seven studies originating in Eastern countries [11-17] was 26.44 cases per 100,000 person-years. The average observed cases of GC in the cohort of GA patients from these seven studies was 461.43 cases per 100,000 person-years. Thus, the pooled SIR for these seven studies was 17.4, with a 95% confidence interval ranging from 15.9 to 19.15 (Table 5).

**Table 5:** Pooled Standardised Incidence Ratios (SIRs) for Gastric Cancer in those with Gastric Atrophy based on region.

Region	Observed Gastric Cancer incidence rate per 100,000 person-years	Expected incidence rate of Gastric Cancer per 100,000 person-years for country of origin of study	Standardised Incidence Ratio	95% CI Lower	95% CI Upper
Eastern	461.43	26.44	17.4	15.9	19.15
Western	204.5	13.8	14.8	12.9	17
Global	304	20	15.1	13.5	16.9

The average expected rate of GC for the 11 studies originating in Western countries [18-28] was 13.8 cases per 100,000 person-years. The average observed cases of GC in the cohort of GA patients from these 11 studies was 204.5 cases per 100,000 person-years. Thus, the pooled SIR for these 11 studies was 14.8, with a 95% confidence interval ranging from 12.9 to 17 (Table 5).

The average global expected rate of GC per 100,000 person-years was 20. The average observed rate of GC in the GA cohort of patients was 304 cases per 100,000 person-years. Thus, the pooled global SIR of GC in those with GA was 15.1, with a 95% confidence interval ranging from 13.5 to 16.9 (Table 5).

Helicobacter pylori (HP) Status of Included Patients and Method for Assessing Degree of GA

HP is a known risk factor for the development of both GA and GC. Four of the included studies did not comment on the HP status of their enrolled patients [15,24-26]. Of the remaining 14 studies, four included only patients in which HP had been eradicated [11,14,18,28], and 10 included mixed cohorts of patients with both HP eradicated and non-eradicated [12,13,16,17,19-23,27].

Twelve of the 18 included studies used the updated Sydney system for assessing the degree of GA, four used the endoscopic assessment system developed by Kimura and Takemoto, one used a modified non-standardised system, and one study did not report what system they used to assess the degree of GA (Table 6).

**Table 6:** Table outlining system used to assess the degree of Gastric Atrophy.

Study ID	System for reporting degree of Gastric Atrophy
Take et al [11]	Kimura and Takemoto
Zhang et al [12]	Updated Sydney System
Song et al [13]	Kimura and Takemoto
Toyoshima et al [14]	Kimura and Takemoto
Sekikawa et al [15]	Kimura and Takemoto
Boreiri et al [16]	Updated Sydney System
Chen et al [17]	Updated Sydney System
Miceli et al [18]	Updated Sydney System
Chapelle et al [19]	Updated Sydney System
Rugge et al [20]	Updated Sydney System
Esposito et al [21]	Updated Sydney System
Hollander et al [22]	Updated Sydney System
Nieminen et al [23]	Updated Sydney System
Dilaghi et al [24]	Not reported
Lahner et al [25]	Updated Sydney System
Ekheden et al [26]	Non-standardised system
Pilozzi et al [27]	Updated Sydney System
Segato et al [28]	Updated Sydney System

Topographical Distribution of GA in Included Studies

Of the 18 included studies, 13 reported the topographical distribution of the GA in their cohort, and five did not (Table 7). Six of the included studies reported the topographical distribution using the OLGA system [29], four reported the distribution using the endoscopic technique described by Kimura and Takemoto [30] and three of the studies reported the distribution using non-validated techniques.

**Table 7:** Table outlining system used to describe the topographical distribution of the Gastric Atrophy. OLGA: Operative Link on Gastritis Assessment

Study ID	Topographical description of Gastric Atrophy
Take et al [11]	Yes, using endoscopic–atrophic-border scale described by Kimura and Takemoto
Zhang et al [12]	Yes
Song et al [13]	Yes, using endoscopic–atrophic-border scale described by Kimura and Takemoto
Toyoshima et al [14]	Yes, using endoscopic–atrophic-border scale described by Kimura and Takemoto
Sekikawa et al [15]	Yes, using endoscopic–atrophic-border scale described by Kimura and Takemoto
Boreiri et al [16]	No
Chen et al [17]	No
Miceli et al [18]	No
Chapelle et al [19]	Yes OLGA System
Rugge et al [20]	Yes OLGA System
Esposito et al [21]	Yes OLGA System
Hollander et al [22]	Yes OLGA System
Nieminen et al [23]	Yes
Dilaghi et al [24]	Yes OLGA System
Lahner et al [25]	Yes OLGA System
Ekheden et al [26]	No
Pilozzi et al [27]	Yes
Segato et al [28]	No

Discussion

This systematic review included a total of 18 studies comprising 25,455 patients. The included studies comprised 10 retrospective cohort studies and eight prospective studies. Seven studies were conducted in Eastern countries, while 11 were conducted in Western countries. Previous studies had demonstrated incidence rates for GC in patients with GA ranging from 1.0 to 15.2 per 1000 person-years [31]. Our study found a pooled incidence rate of 1.7 cases per 1000 person-years with a 95% confidence interval of 1.5 to 1.9. We performed a subgroup analysis by grouping studies based on their geographical location into western and eastern cohorts; this identified an incidence rate of one case per 1000 person-years with a 95% confidence interval of 0.9 to 1.2 for the cohort of Western countries and an incidence rate of 3.6 cases of GC per 1000 person-years with a 95% confidence interval of 3.2 to 4.2 in the eastern cohort. This would support previous findings that the overall incidence rate of GC in Eastern countries is higher than in the West [32].

However, when we explored the relative risk (RR) of GC in those with GA, using SIRs as a measure of RR, we found a pooled global SIR of 15.1 with a 95% confidence interval ranging from 13.5 to 16.9. This is higher than previously reported and is also higher than the reported relative risk of adenocarcinoma among patients with non-dysplastic Barrett’s oesophagus [33].

When we conducted a subgroup analysis based on the country of origin of the study, grouping studies again into Western and Eastern cohorts, we found SIRs of 14.8 with a 95% confidence interval ranging from 12.9 to 17 for the cohort of Western countries and 17.4 with a 95% confidence interval ranging from 15.9 to 19.15 for the eastern cohort which again is higher than previously reported and higher compared to the relative risk for developing adenocarcinoma in non-dysplastic Barrett’s oesophagus.

This would indicate that the risk of developing intestinal-type gastric cancer may be higher than previously appreciated in patients with GA. However, this study has several limitations, so the results must be interpreted cautiously. The limitations of this study highlight the overall heterogeneity of studies being conducted in this area.

Our review included 18 studies; however, 10 were retrospective and thus prone to increased bias. The number of patients enrolled in the studies was variable, with a low of five and a high of 14285.

The role of H. pylori in the pathogenesis of the Correa cascade is well described, namely in the initiation of the chronic gastritis phase. However, four of the included studies did not comment on the HP status of their enrolled patients, and 10 included mixed cohorts or cohorts in whom the H. pylori status was not known. This limited our ability to perform subgroup analysis to assess the effect of H. pylori on the risk of GA progressing to GC.

Furthermore, the gold standard for diagnosing gastric atrophy is based on histological assessment; however, four of the included studies used the endoscopic assessment system developed by Kimura and Takemoto. Although this has been previously demonstrated to have similar diagnostic accuracy to histological assessment in diagnosing GA, it requires experienced endoscopists and thus may not be reproducible.

A further critical determinant of the risk of GA progressing to GC is the topographical distribution of the GA. Antral predominant GA is considered to have a lower risk of progression compared to a pan-gastric GA. This fact is recognised in the guideline documents from the British Society of Gastroenterology, The American Gastroenterological Association and the European Society of Gastrointestinal Endoscopy in their MAPS 2 guideline document [34-36]. Five of the studies in this review did not report the topographical distribution of the GA in their cohort. Of the 13 that did, four reported the distribution using the endoscopic technique proposed by Kimura and Takemoto as previously described. This technique is operator-dependent and may not be reproducible. Three of the studies reported the topographical distribution using non-validated techniques.

Overall, our study has highlighted several points. The risk of progression of GA to GC may be higher than previously appreciated. This may not have significant implications in countries with endoscopic screening protocols, but it would have substantial implications for lower-risk Western countries where routine upper endoscopy surveillance is not performed. If the risk of progression is higher than that seen in non-dysplastic Barrett’s, then there may indeed be an argument for increased investment in endoscopic surveillance programs. However, arguably, the most significant finding in our review was the level of heterogeneity in the studies being conducted in this area. Future studies must be rigorous and ensure that the key contributing factors such as H. pylori status, topographical distribution and GA assessment techniques are controlled for.

## Conclusions

GA does increase the risk of developing GC, and this risk may be higher than previously appreciated. Further large-scale studies are needed in Western cohorts of patients to precisely define this risk and guide the need for surveillance programs. These future studies must be standardised to account for H. pylori status, the topographical distribution of the GA, and the methods for assessing the degree of GA.
